# Efficacy and safety of primary suture following laparoscopic common bile duct exploration in the treatment of elderly patients with cholecystolithiasis complicated by choledocholithiasis

**DOI:** 10.3389/fsurg.2025.1683506

**Published:** 2025-12-08

**Authors:** Huixing Li, Xuhong Duan, Zhenyu Wu

**Affiliations:** 1 Department of Hepatobiliary Surgery, Aerospace Center Hospital, Beijing, China; 2 Department of Gastroenterology, First Medical Center, General Hospital of PLA, Beijing, China

**Keywords:** choledocholithiasis, laparoscopic common bile duct exploration, primary suture, T-tube drainage, elderly patients

## Abstract

**Background and aim:**

The study aimed to evaluate the efficacy and safety of primary suture following laparoscopic common bile duct exploration (LCBDE) in the treatment of elderly patients with cholecystolithiasis complicated by choledocholithiasis.

**Methods:**

We retrospectively reviewed 164 elderly patients, aged older than 70 years, who underwent laparoscopic cholecystectomy (LC) and laparoscopic common bile duct exploration at our center from January 2015 to December 2024. The patients were categorized into two groups according to the surgical strategy used, namely, primary suture following LC + LCBDE (PS group) and T-tube drainage following LC + LCBDE (T-tube group). General data, intraoperative and postoperative outcomes, and postoperative complications in the two groups were compared and analyzed.

**Results:**

There were no significant differences in age (77.1 ± 4.6 vs. 78.3 ± 5.2 years, *p* = 0.126), gender (*p* = 0.523), body mass index (24.7 ± 2.1 vs. 24.3 ± 1.8, *p* = 0.192), diameter of common bile duct (14.1 ± 3.8 vs. 13.4 ± 2.5 mm, *p* = 0.158), American Society of Anesthesiologists risk grading (*p* = 0.545), and hematological indicators and comorbidities (*p* > 0.05) between the two groups. All the patients successfully underwent laparoscopic surgery without any case being converted to laparotomy. There was no significant difference in intraoperative blood loss between the PS and T-tube groups (54.6 ± 26.4 vs. 58.8 ± 24.7 mL, *p* = 0.297). However, surgical duration (113.7 ± 23.8 vs. 129.2 ± 39.5 min, *p* = 0.004) was significantly shorter in the PS group. In addition, the PS group had a significantly shorter exhaustion time (2.1 ± 0.7 vs. 2.9 ± 0.6 days, *p* = 0.000) and postoperative hospital stay (6.1 ± 1.9 vs. 8.2 ± 1.6 days, *p* = 0.000) and lower hospitalization expenses (21.2 ± 5.9 vs. 26.6 ± 7.4 thousand, *p* = 0.000). In terms of postoperative complications, there were significant differences in the incidences of electrolyte disturbance (11.3% vs. 23.7%, *p* = 0.042) and bile leakage (0.0% vs. 8.6%, *p* = 0.030) between the PS and T-tube groups. No serious complications, such as intra-abdominal infection, bleeding, or death, occurred in either group.

**Conclusion:**

Primary suture following LCBDE is a safe and effective treatment approach for cholecystolithiasis combined with choledocholithiasis in elderly patients. When the surgical criteria are strictly executed, primary suture offers superior perioperative outcomes over T-tube drainage, facilitating patients’ recovery without increasing postoperative complications.

## Introduction

Choledocholithiasis is one of the most common diseases in the world and is present in approximately 10%–15% of individuals with cholecystolithiasis (1, 2). Furthermore, the incidence of choledocholithiasis increases with age and is especially high in elderly patients aged older than 70 years (3–5). This disease is the result of stones expelled from the gallbladder or intrahepatic bile duct. Small stones can pass into the duodenum via the major duodenal papilla without any complications; however, larger stones may cause obstructions due to their size, which can subsequently lead to complications such as acute cholangitis, obstructive jaundice, hepatic dysfunction, and acute pancreatitis (6–8). The conventional treatment for patients with cholecystolithiasis complicated by choledocholithiasis is T-tube drainage following open cholecystectomy combined with exploration of the common bile duct (CBD). In recent decades, with the development of minimally invasive concepts, laparoscopic and endoscopic surgery have become increasingly popular, including laparoscopic cholecystectomy and laparoscopic common bile duct exploration (LC + LCBDE) and laparoscopic cholecystectomy followed by endoscopic retrograde cholangiopancreatography (ERCP + LC). It should be noted that the ERCP + LC approach is usually combined with endoscopic sphincterotomy (EST), and EST tends to cause sphincter of Oddi dysfunction, which is linked to recurrent cholangitis and long-term reflux of duodenal contents (9–11). Moreover, complications such as pancreatitis, bleeding, and perforation caused by ERCP limit its clinical efficacy (12, 13). The LC + LCBDE strategy has therefore become an essential treatment for cholecystolithiasis complicated by choledocholithiasis, with advantages such as leaving a small wound and rapid postoperative recovery, especially for elderly patients. Moreover, with the continuous improvement of laparoscopic operation techniques, it also has a high success rate.

Traditionally, LCBDE followed by T-tube drainage has been widely used to support bile duct decompression and drainage, avoid possible bile leakage, and retain an approach postoperatively for further choledochoscopy to remove any residual stones (14–17). However, T-tube drainage also has some disadvantages, such as decreased quality of life, drainage site pain, difficulties associated with nursing care, electrolyte disturbance due to bile loss, T-tube dislocation, and repeated postoperative examinations (15, 18–20). Furthermore, the T-tube needs to be carried for months before removal, which increases the level of inconvenience for the patient and negatively impacts their quality of life (19, 21). These disadvantages are more likely to occur in elderly patients due to their fragile physical condition and decreased cognitive ability. Consequently, clinicians attempt to directly suture the CBD incision to preserve its integrity, restoring normal physiological function, facilitating early resumption of gastrointestinal activities, enhancing postoperative recovery, shortening the length of hospital stay, and improving quality of life (3–22). Moreover, this technique does not require a second hospitalization for T-tube removal, and is thereby significantly more convenient for the patient and reduces cost (23). With the improved techniques of laparoscopic suturing and knotting, LCBDE with choledochotomy primary closure has become a widely recognized surgical strategy. In recent years, studies have shown that primary suture following LCBDE is a safe and feasible technique for eligible patients and is superior to T-tube placement (3, 20, 23). However, few studies on elderly patients have been reported. Our center has performed the primary suture technique following LCBDE for decades, and a proportion of the patients were elderly people older than 70 years. This study aimed to evaluate the efficacy and safety of primary suture following laparoscopic common bile duct exploration in the treatment of patients aged ≥70 years, to provide more evidence for selecting the optimal surgical strategy in the treatment of elderly patients with cholecystolithiasis complicated by choledocholithiasis.

## Materials and methods

### Patients

This was a retrospective study that compared primary suture following LC + LCBDE and T-tube drainage following LC + LCBDE. The inclusion criteria were as follows: (1) a definitive preoperative diagnosis of cholecystolithiasis complicated with choledocholithiasis, (2) patients who successfully underwent the LC + LCBDE surgery, (3) aged ≥70 years, and (4) complete and searchable medical records. The exclusion criteria were as follows: (1) aged <70 years, (2) systemic infections, (3) presence of malignant tumors, (4) intrahepatic bile duct stones, (5) biliary malformation or distal common bile duct stricture, (6) conversion to laparotomy, and (7) incomplete patient clinical data. This study received approval from the Ethics Committee of the Aerospace Center Hospital. Informed consent was obtained from all the patients and their families. According to the inclusion criteria, 164 patients who were hospitalized in the Department of Hepatobiliary Surgery at the Aerospace Center Hospital between January 2015 and December 2024 were enrolled. All the patients were diagnosed preoperatively by imaging modalities, such as abdominal ultrasound, computed tomography (CT), and/or magnetic resonance cholangiopancreatography (MRCP). The patients were divided into two groups according to surgical method, namely, primary suture following LC + LCBDE (PS group, *n* = 71) and T-tube drainage following LC + LCBDE (T-tube group, *n* = 93). All the surgeries were performed by experienced surgeons, and the decision between the two approaches depended on the surgeon's judgment according to the patient’s situation or the patient’s self-selection after informed consent. Our criteria for primary suture of the CBD were as following: (1) diameter of CBD ≥ 10 mm, (2) no residual stones after completely removal stones confirmed through cholangioscopy, (3) mild inflammation of the CBD wall and no distal common bile duct stricture, (4) well-functioning sphincter of Oddi, and (5) no severe injuries to the supplying vessels of the CBD. Patients who were deemed unsuitable for primary closure met the criteria for T-tube insertion. Meanwhile, additional examinations, namely, echocardiography and a pulmonary function test, were routinely performed to fully exclude contraindications for laparoscopic surgery and general anesthesia.

### Surgical procedures

**Figure 1:**
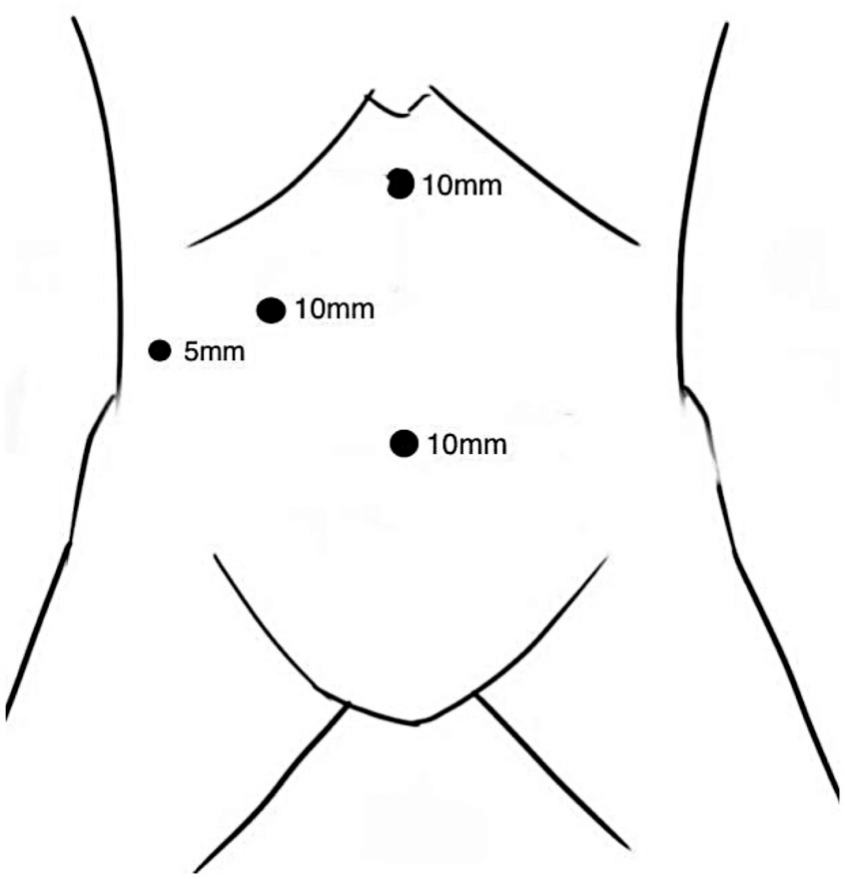
The schematic diagram of the trocar placement.

Under satisfactory general endotracheal anesthesia, patients were positioned in the reverse Trendelenburg position and the operating table was tilted to the left by 30° to facilitate exposure and comfortable operation. CO_2_ pneumoperitoneum with a pressure of 13 mmHg (1 mmHg = 0.133 kPa) was established by puncturing the infraumbilical region with a Veress needle. The four-hole technique was used routinely. One trocar (10 mm) was placed infra-umbilically to serve as the laparoscopic observation hole, one trocar (10 mm) was placed 3–4 cm below the xiphoid process to serve as the main operating hole, one trocar (10 mm) was placed at 5 cm below the right subcostal midline of the clavicle, and one trocar (5 mm) was placed 7 cm below the rib margin of the right axillary line to serve as an auxiliary operation hole (Figure 1).

LC was routinely performed first. After dissecting the Calot triangle using electrocautery, the cystic duct and cystic artery were exposed and the CBD was identified simultaneously. The cystic artery was clamped with ligation clips and then disconnected. The cystic duct was clamped with ligation clips but not disconnected temporarily, and the gallbladder was retrogradely freed by electrocoagulation from the gallbladder bed. The cystic duct was pulled to expose the CBD, and then the serosa was freed from the CBD's anterior wall. The anterior wall of the CBD was incised approximately 1.0 cm longitudinally using electrocautery in the upper portion of the duodenum, and a cholangioscope (Olympus Corp., Shinjuku, Tokyo, Japan) was then inserted into the CBD through the main operating port for bile duct exploration and stone removal. The stones were removed using a stone extractor (Cook Medical, Bloomington, IN, USA). If a stone was too large to be removed, the incision was appropriately extended. After completely removing the stones, the biliary tract was flushed with normal saline and repeatedly inspected with cholangioscopy. We then ensured that there were no residual stones and the function of the sphincter of Oddi was normal, which could be seen clearly and completely opening or closing with the change in biliary pressure caused by normal saline flushing. We then completely removed the gallbladder after disconnecting the cystic duct, and removed the gallbladder and stones from the abdominal cavity through the main operating port after placing them in a retrieval bag. The surgical area was rinsed with normal saline thoroughly to ensure there was no bleeding or residual stones before proceeding to the next procedure, namely, inserting a T-tube drain or primary suturing of the incision in the CBD. It should be noted that if cholangioscopy revealed severe inflammation of the CBD wall or the function of the sphincter of Oddi was abnormal, we inserted a T-tube drain even though primary suture was planned preoperatively.

**Figure 2:**
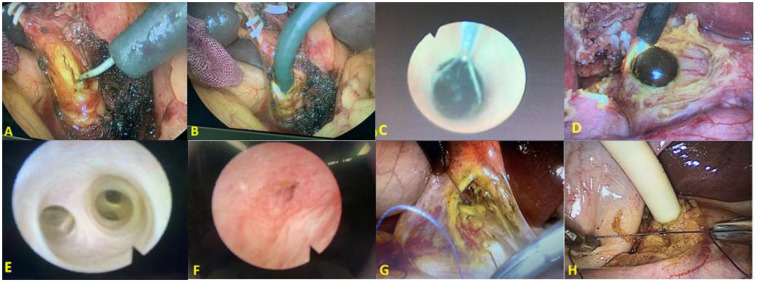
The main surgical procedures: incising the anterior wall of CBD using electrocautery (**A**); inserting a cholangioscope into the CBD (**B**); a stone is removed using a stone extractor (**C,D**); exploring the intrahepatic bile ducts (**E**); exploring the distal end of the CBD and observing the function of the duodenal papilla (**F**); primary suturing (**G**); and inserting a T-tube (**H**).

In the T-tube group, a silicone T-tube (20 Fr) was trimmed to the appropriate size and inserted into the CBD. The 10 mm trocar below the xiphoid process was routinely used as the main operating hole, and the laparoscopic needle holder was inserted into the abdominal cavity through this trocar to suture the CBD. Then, 4-0 absorbable sutures (Ethicon, Inc., Somerville, NJ, USA) were used to close the incision in the CBD using an intermittent everting suture technique. It is worth noting that we were careful not to suture the T-tube to the bile duct wall during the suturing process. After suturing, the T-tube was pulled out of the abdominal cavity through the trocar at the right subcostal midline of the clavicle. We then observed whether there was any liquid leakage at the edge of the T-tube by injecting 30–50 mL of normal saline into it. If there was any liquid leakage, we sutured the leakage point and tested again until there was no liquid exudation. In the PS group, the incision in the CBD was sutured intermittently using 4-0 absorbable sutures, with a margin of approximately 1.0 mm and a needle pitch of approximately 2 mm. After completely suturing the incision, we observe the incision for a while to check whether there was bile leakage. Intermittent sutures were added at the leakage points when they were found. Before the end of the operation, the surgical area was repeatedly flushed with normal saline to ensure there was no bile leakage or bleeding. A peritoneal cavity drainage tube was then routinely inserted through the foramen of Winslow and pulled out of the abdominal cavity through the trocar hole at the rib margin of the right axillary line. The surgical procedures are shown in Figure 2.

### Postoperative management

After the operation, the patients were routinely given symptomatic treatment, including liver protection, anti-inflammation, acid suppression, and nutrition support by intravenous infusion for 3 days. The gastric and urinary tubes were removed within 24 h after the operation, and the patients were instructed to eat a liquid diet and perform appropriate activities to promote the recovery of gastrointestinal function and prevent venous thrombosis of the lower limbs. They were then gradually moved to a semi-liquid diet and then a normal diet. Blood tests, biochemistry, and coagulation function were routinely rechecked every 3 days postoperatively to monitor the recovery of the patients. Ultrasound of the lower limb blood vessels was routinely performed 24 h after the operation to check for the presence of lower limb venous thrombosis. If it revealed lower limb venous thrombosis, low-molecular-weight heparin was administered to prevent its progression. The color and amount of fluid in the drainage tube were observed, and the drainage tube was removed 48–72 h postoperatively if the amount of fluid in the drainage tube was less than 30 mL/day and no postoperative bile leakage was observed. The patients were discharged from the hospital if there were no abnormalities in the biochemical tests and imaging examinations. The patients in the T-tube group were advised to clamp the T-tube 2 weeks postoperatively. If there was no significant abdominal discomfort, the T-tube remained clamped. The patients were readmitted 8 weeks after discharge. T-tube angiography was performed to confirm the absence of residual stones before T-tube removal, and cholangioscopy was simultaneously performed through the T-tube sinus tract to further confirm the absence of residual stones in the biliary tract. The patients in the PS group were advised to undergo a repeat MRCP to check for residual biliary stones and biliary stricture 4 weeks postoperatively.

### Follow-up

Researchers from the Department of Hepatobiliary Surgery at the Aerospace Center Hospital followed up with the patients by telephone and outpatient visits.

### Data collection

Preoperative data: gender, age, liver function indices, electrolytes, white blood cell (WBC) count, diameter of the CBD, body mass index (BMI), American Society of Anesthesiologists (ASA) class, and comorbidities.

Intraoperative data: operation time and estimated blood loss.

Postoperative data: anal exhaustion time, liver function indices, electrolytes, WBC count, complications, hospital stay, and hospitalization expenses.

Follow-up parameters: stone recurrence, bile duct stricture, accident events, physical condition, and satisfaction with the quality of life.

### Statistical analysis

All the variables were analyzed using SPSS 20.0 software. Continuous variables are expressed as means ± SD and t-tests were used to compare the groups. Categorical variables were tested using the *χ*^2^ test and Fisher's exact test. *p* < 0.05 was considered statistically significant.

## Results

### Preoperative data

A total of 164 eligible patients were enrolled, including 71 patients in the PS group and 93 patients in the T-tube group. All patients’ choledocholithiases were primary stones, and there was no previous surgical history of choledocholithiasis. There were no significant differences in age, gender, BMI, liver function indices, electrolytes, WBC count, diameter of CBD, ASA class, and comorbidities between the two groups (Table 1).

**Table 1:** General characteristics of the patients.

Index	PS group (*n* = 71)	T-tube group (*n* = 93)	*p*-value
Age (years)	77.1 ± 4.6	78.3 ± 5.2	0.126
Gender			
Male, *n* (%)	40 (56.3)	57 (61.3)	0.523
Female, *n* (%)	31 (43.7)	36 (38.7)	
BMI (kg/m^2^)	24.7 ± 2.1	24.3 ± 1.8	0.192
WBC (10^9^/L)	11.3 ± 1.6	11.8 ± 2.4	0.131
TBIL (μmol/L)	85.4 ± 19.7	90.3 ± 25.1	0.177
DBIL (μmol/L)	55.2 ± 11.4	58.3 ± 13.5	0.122
ALT (U/L)	185.7 ± 41.8	197.2 ± 52.2	0.130
AST (U/L)	226.8 ± 51.4	237.3 ± 65.8	0.269
ALP (U/L)	165.9 ± 24.2	172.5 ± 30.7	0.138
K^+^ (mmol/L)	4.5 ± 0.6	4.6 ± 0.3	0.165
Na^+^ (mmol/L)	139.2 ± 3.1	138.6 ± 2.8	0.196
Cl^−^ (mmol/L)	101.7 ± 2.3	101.9 ± 1.9	0.524
Diameter (mm)	14.1 ± 3.8	13.4 ± 2.5	0.158
ASA grading, *n* (%)			
I	13 (18.3)	15 (16.1)	0.545
II	56 (78.9)	72 (77.4)	
III	2 (2.8)	6 (6.5)	
Comorbidities, *n* (%)			
Diabetes mellitus	32 (45.1)	38 (40.8)	0.589
Hypertension	55 (77.5)	67 (72.0)	0.430
Cardiovascular disease	18 (25.4)	29 (31.2)	0.413
Others	9 (12.7)	14(15.1)	0.664

BMI, body mass index; WBC, white blood cell; TBIL, total bilirubin; DBIL, direct bilirubin; ALT, alanine aminotransferase; AST, aspartate aminotransferase; ALP, alkaline phosphatase; ASA, American Society of Anesthesiologists.

### Intraoperative outcomes

All the patients successfully underwent laparoscopic surgery without any case being converted to laparotomy. Eight cases in the T-tube group were preoperatively planned to undergo primary suture, but changed to T-tube drainage due to the intraoperative discovery of severe inflammation of the CBD wall or abnormal function of the sphincter of Oddi through cholangioscopy. There was no significant difference in intraoperative bleeding between the PS and T-tube groups [(54.6 ± 26.4) mL vs. (58.8 ± 24.7) mL, *p* = 0.297]. However, a significant difference was observed in surgical duration between the PS and T-tube groups [(113.7 ± 23.8) min vs. (129.2 ± 39.5) min, *p* = 0.004].

### Postoperative outcomes and complications

In terms of postoperative outcomes, no significant differences were shown in liver function indices and WBC count. However, the PS group exhibited superior performance compared to the T-tube group. Specifically, the PS group had significantly shorter exhaustion time (2.1 ± 0.7 vs. 2.9 ± 0.6 days, *p* = 0.000) and postoperative hospital stay (6.1 ± 1.9 vs. 8.2 ± 1.6 days, *p* = 0.000), and lower hospitalization expenses (21.2 ± 5.9 vs. 26.6 ± 7.4 thousand, *p* = 0.000). Moreover, in terms of postoperative complications, the incidence of hyperamylasemia (23.9% vs. 16.1%) and lower limb venous thrombosis (11.3% vs. 20.4%) between the PS and T-tube groups showed no significant difference. However, there were significant differences in the incidences of electrolyte disturbance (11.3% vs. 23.7%, *p* = 0.042) and bile leakage (0.0% vs. 8.6%, *p* = 0.030) between the PS and T-tube groups (Table 2). Among the eight cases of bile leakage in the T-tube group, five cases were caused by T-tube folding in the abdominal cavity and three cases were caused by the T-tube prolapsing from the bile duct. All were confirmed by T-tube angiography. All these cases were solved by adjusting the T-tube's location during angiography or a second laparoscopic surgery to re-insert the T-tube and re-suture. None of the cases deteriorated further. All the other complications were managed effectively with symptomatic treatments, including enzyme inhibition, anti-coagulation, and electrolyte supplementation. No serious complications, such as intra-abdominal infection, bleeding, or death, occurred in either group.

**Table 2:** Postoperative outcomes and complications.

Index	PS group (*n* = 71)	T-tube group (*n* = 93)	*p*-value
WBC (10^9^/L)	7.3 ± 2.6	6.8 ± 1.4	0.116
TBIL (μmol/L)	29.7 ± 18.2	33.8 ± 19.6	0.173
DBIL (μmol/L)	18.9 ± 7.4	20.3 ± 9.1	0.292
ALT (U/L)	82.7 ± 49.6	78.6 ± 36.8	0.544
AST (U/L)	61.4 ± 35.2	54.9 ± 31.5	0.215
ALP (U/L)	85.6 ± 31.7	90.7 ± 29.3	0.288
Exhaustion time (days)	2.1 ± 0.7	2.9 ± 0.6	0.000
Postoperative hospital stay (days)	6.1 ± 1.9	8.2 ± 1.6	0.000
Hospitalization cost (thousand)	21.2 ± 5.9	26.6 ± 7.4	0.000
Complications, *n* (%)			
Hyperamylasemia	17 (23.9)	15 (16.1)	0.211
Electrolyte disturbances	8 (11.3)	22 (23.7)	0.042
Bile leakage	0 (0.0)	8 (8.6)	0.030
Lower limb venous thrombosis	8 (11.3)	19 (20.4)	0.117

WBC, white blood cell; TBIL, total bilirubin; DBIL, direct bilirubin; ALT, alanine aminotransferase; AST, aspartate aminotransferase; ALP, alkaline phosphatase.

All hematological indicator values were taken from the test results on the third day after surgery.

### Follow-up

All the patients were followed up for 2–24 months postoperatively, with a follow-up rate of 100%. There were no cases of stone recurrence, bile duct stricture, or death in either group. In total, 10 cases in the T-tube group accidentally pulled their T-tube out before removal. After emergency admission, they underwent cholangioscopy to check the status of the bile duct. Due to the good formation of the T-tube sinus tracts, there was no bile leakage into the abdominal cavities in any of these cases, and no bleeding or residual stones were found in the biliary tracts. They were treated with pain relief and systemic fluid infusion, and discharged after improvement in their physical condition. Furthermore, 26 cases in the T-tube group were dissatisfied with their postoperative quality of life due to factors such as discomfort when carrying the T-tube, intermittent pain at the fixation site of the T-tube, intermittent dressing changes, nursing related to the T-tube, and decreased appetite. In contrast, the patients in the PS group were generally satisfied with their postoperative quality of life, and no residual stones or biliary stricture were found by MRCP examinations. Therefore, the treatment satisfaction rate of patients in the PS group was significantly higher than that of the T-tube group (100.0% vs. 72.0%, *p* = 0.000).

## Discussion

With the continuous development of laparoscopic surgery and the maturation of laparoscopic techniques, LC + LCBDE has become the mainstream surgical method for the treatment of cholecystolithiasis complicated by choledocholithiasis. Compared with laparotomy and ERCP + EST, this procedure has the advantages of resulting in less trauma and no damage to the normal structure of the duodenal papilla, and avoiding postoperative complications such as acute pancreatitis, retrograde biliary infection, and bleeding (9). However, CBDE also results in some postoperative complications such as bile leakage, biliary stricture, and residual stones. Thus, an indwelling T-tube has been routinely placed in the CBD during this surgery for bile drainage and biliary decompression, providing ductal stenting and preventing biliary stricture. Moreover, it also maintains access for postoperative cholangiography and choledochoscopic stone removal. After prolonged clinical application, several drawbacks to T-tube drainage have gradually been revealed, which are as follows: (1) accidental T-tube dislodgement can result in biliary peritonitis and surgical intervention; (2) prolonged bile drainage can badly affect a patient’s digestive function and lead to electrolyte disturbance; (3) postoperative T-tube retention routinely lasts 6–8 weeks, which increases the nursing workload, imposing a significant psychological burden on the patient and negatively impacting their quality of life; and (4) predisposition to inflammatory stenosis, which is attributed to the chronic mechanical irritation of the biliary epithelium caused by long-term T-tube placement. These complications are more likely to occur in elderly patients due to their decreased physical function and cognitive ability. Reduced digestive function makes them more susceptible to the effects of bile loss, and decreased physical activity and cognitive ability make them more prone to accidental T-tube dislodgement, which could impose pain and a psychological burden on them.

Hence, some clinicians have proposed the alternative surgical method of primary suture after LCBDE when treating eligible patients with choledocholithiasis. Compared to T-tube drainage, this technique can preserve the integrity of the CBD and reduce bile loss, which further facilitates the quicker recovery of digestive function, shortens hospitalization time, and reduces hospitalization costs. However, there is still controversy over this technique concerning the increase in postoperative biliary pressure, which may lead to bile leakage, serious biliary peritonitis, and a higher risk of biliary stricture. Furthermore, it is not as easy to deal with early postoperative biliary residual stones when using this technique compared with an indwelling T-tube (4). Over the past few years, a series of studies comparing primary suture and T-tube drainage after LCBDE have been published. According to these studies, primary suture of the CBD after exploration is a safe and effective method for treating eligible patients with choledocholithiasis. Dong et al. (24) conducted a prospective study that involved 194 patients who underwent surgery for CBD stones, comparing the outcomes between primary suture in 101 patients and T-tube drainage in 93 patients. The results indicated that primary suture was superior in reducing operation time, the time to recovery of intestinal function, the length of postoperative hospital stay, and hospitalization costs. As earlier bile flow back to the intestine after primary suture promotes quicker intestinal peristalsis, patients can resume eating sooner, and the reduction in operation time decreases the risk of postoperative thrombosis and respiratory or cardiac complications. This is particularly beneficial for elderly patients with cardiovascular or respiratory risks. Furthermore, primary suture does not require T-tube cholangiography and hospital re-admissions for T-tube removal, thus reducing overall hospitalization costs. In addition, there were no significant differences in the incidence of postoperative complications, such as bile leakage and residual stones, between the two groups. The long-term research results indicated that the patients who underwent primary suture had low biliary stricture and recurrence rates.

Currently, the incidence of choledocholithiasis is especially high in elderly patients who have coexisting chronic diseases and risk factors, such as cardiopulmonary disease, diabetes mellitus, and worse ASA physical status, which causes them to be more susceptible to morbidity and mortality. However, with the rapid development of laparoscopic skills and perioperative management, including a comprehensive preoperative evaluation of cardiopulmonary function, precise anesthesia management during surgery, and careful postoperative care, it has been established that age no longer represents a contraindication to laparoscopic biliary surgery. Surgeons have successfully carried out LCBDE in elderly patients and gradually accumulated extensive experience (25–28). In this study, we compared the efficacy and safety of primary suture vs. T-tube drainage following LCBDE in elderly patients. The results clearly demonstrated that primary suture is a safe and effective technique with shorter operation times, shorter exhaustion times, shorter postoperative hospital stay, and lower hospitalization costs, and resulted in a lower incidence of postoperative complications, such as electrolyte disturbance and bile leakage. We further analyzed the reasons for such differences. First, the elimination of the intraoperative placement of a T-tube and the suturing of the incision in the bile duct reduces the operation time. Furthermore, the reduction of bile loss facilitates quicker recovery of digestive function and accelerates the patient’s recovery, which could further reduce the hospitalization time and hospitalization costs. Moreover, the technique does not require a second hospitalization for T-tube removal, thereby significantly improving patient convenience and reducing costs. As for postoperative complications, the reduction in bile loss is also a key point. The main components of bile include bile acid salts, bile pigments, cholesterol, phospholipids, and electrolytes. Its function is to emulsify fat, promote lipase digestion of fat, assist in lipid absorption, and promote the absorption of fat-soluble vitamins. Thus, the reduction in bile loss is beneficial for the absorption of nutrients in the intestine and maintains electrolyte balance.

Bile leakage is a major criterion for assessing the safety of primary suture following LCBDE for the treatment of choledocholithiasis. In our study, no bile leakage was observed in the PS group; however, eight cases (8.6%) in the T-tube group experienced postoperative bile leakage, of which five cases were caused by the T-tube folding in the abdominal cavity and three cases were caused by the T-tube prolapsing from the bile duct. All were confirmed by T-tube angiography. All these cases were solved by adjusting the T-tube's location during angiography or a second laparoscopic surgery to re-insert the T-tube and re-suture. None of the cases deteriorated further. There was a significant difference in the incidence of bile leakage between the two groups. We argue that the proficient execution of laparoscopic suturing techniques and precise suturing are key to avoiding postoperative bile leakage. Therefore, all the surgeries were performed by surgeons with more than 10 years of experience in our department, and the incisions were carefully checked for any bile leakage after suturing. Intermittent sutures were performed at any leakage points when they were found. There is almost no postoperative bile leakage when the criteria for primary closure are strictly executed. However, the insertion of a T-tube increases the possibility of it folding or prolapsing from the bile duct, thereby increasing the possibility of postoperative bile leakage. Some studies have also shown that T-tube drainage does not prevent postoperative bile leakage (8, 21, 29, 30). The poor mobility and self-care ability of elderly patients often lead them to accidentally pull the T-tube, which could further lead to pain and T-tube dislodgement. Concern about such accidents also imposes a heavy psychological burden on them. Some patients forget to clamp the T-tube postoperatively, resulting in long-term loss of bile, which seriously affects their appetite and electrolyte balance. Postoperatively, these drawbacks inconvenience patients, seriously affecting their quality of life and causing some patients to be dissatisfied with their postoperative quality of life. However, primary suture following LCBDE completely eliminates patients’ concerns about these issues, which could greatly improve their satisfaction with their postoperative quality of life. In our study, the patients’ postoperative satisfaction rate in the PS group was 100%, which was significantly higher than the 72.0% in the T-tube drainage group.

Although primary suture following LCBDE is significantly better than T-tube drainage, we should note that not all patients are suitable for primary suture. T-tube drainage following LCBDE is still a necessary treatment strategy for choledocholithiasis. Selecting eligible patients and strictly executing the criteria of primary suture can enable patients to undergo an optimized therapeutic schedule and reduce the incidence of postoperative complications. In our study, eight cases were planned to undergo primary suture preoperatively, but were changed to T-tube drainage due to the intraoperative discovery of severe inflammation of the CBD wall or poor functioning of the sphincter of Oddi through cholangioscopy. Certain limitations of this study should be acknowledged. As it was a retrospective study, there may have been some selection bias in patient grouping, which would have had an impact on the accuracy of the results of this study. We will continue to conduct prospective large-scale studies in this field to provide more accurate and effective research results for clinical practice.

## Conclusion

In conclusion, primary suture following LCBDE is a safe and effective treatment approach for cholecystolithiasis combined with choledocholithiasis in elderly patients. When the surgical criteria are strictly executed, primary suture offers superior perioperative outcomes compared to T-tube drainage, facilitating patients’ recovery without increasing postoperative complications.

## Data Availability

The raw data supporting the conclusions of this article will be made available by the authors, without undue reservation.
